# Co‐Coverage of Social Protection Programs and Maternal and Child Nutrition Interventions in Bangladesh, India and Nepal

**DOI:** 10.1111/mcn.70233

**Published:** 2026-09-01

**Authors:** Samuel Scott, Sumanta Neupane, Hana Tasic, Nadia Akseer, Sunny S. Kim, Harold Alderman, Bianca Carducci, Rebecca Heidkamp

**Affiliations:** ^1^ International Food Policy Research Institute Washington, DC USA; ^2^ International Food Policy Research Institute Kathmandu Nepal; ^3^ Modern Scientists Global Ontario Canada; ^4^ Johns Hopkins University Baltimore USA; ^5^ Johns Hopkins Bloomberg School of Public Health Baltimore, Maryland USA

**Keywords:** children, co‐coverage, health system, nutrition, social protection, women

## Abstract

Social protection programs (SPPs) are commonly used in South Asia to address inequities in meeting basic needs. Provision of both SPP benefits and essential health/nutrition interventions to mothers and children is a goal toward optimal health and development outcomes, but co‐coverage of SPPs and health/nutrition interventions among beneficiary households is poorly described. Using six population‐based surveys in Bangladesh, India, and Nepal (*n* = 253,703 women with children under 5 years of age), we examined data availability for SPPs (food and cash transfers) and health/nutrition interventions, and estimated their coverage and co‐coverage during a woman's last pregnancy (interventions: take‐home food rations plus at least four antenatal care visits, receipt of iron‐folic acid tablets, deworming, and tetanus injections), after delivery (cash benefit plus the interventions above), and in children (take‐home ration for the child plus vitamin A supplementation, deworming, iron syrup, growth monitoring, and nutrition counseling). In India, 52% and 51% of women and children, respectively, received food transfers, but only 3% and 8% received food plus all health/nutrition interventions. In India and Nepal, respectively, cash after delivery was received by 41% and 86% of women, but only 4% and 22% received cash after delivery plus all health/nutrition interventions. There were insufficient data to estimate co‐coverage in Bangladesh, which only had data on cash/food transfers but not on health/nutrition interventions. Our findings highlight the need for data on both SPP and health/nutrition intervention coverage in household surveys to be able to estimate co‐coverage. Estimates of co‐coverage help identify missed opportunities to reach women and children with interventions across multiple sectors.

## Summary

- In countries with multiple programs addressing basic needs, it is important to assess whether they are reaching their intended beneficiaries.
- Receipt of social protection benefits (cash and/or in‐kind transfers) and nutrition interventions can improve health and development outcomes among vulnerable groups, including mothers and young children.
- Using publicly available, population‐based survey data, we assessed the feasibility of estimating the concurrent reach (co‐coverage) of social protection and health and nutrition interventions in Bangladesh, India, and Nepal.
- Limited availability of program data in household surveys constrains co‐coverage estimation; where data exist, co‐coverage of social protection and maternal and child health and nutrition interventions is generally low.
- Multitopic household surveys should incorporate measures that enable routine monitoring of the concurrent reach of programs designed to address households' multiple needs.

**Abbreviations**
ANC: antenatal care
BMICS: Bangladesh Multiple Indicator Cluster Survey
CCT: conditional cash transfer
DHS: Demographic and Health Survey
HIES: Household Income and Expenditure Survey
ICDS: Integrated Child Development Services
IFA: iron folic acid
NDHS: Nepal Demographic and Health Survey
NFHS: National Family Health Survey
NMICS: Nepal Multiple Indicator Cluster Survey
NSS: National Sample Survey
SPP: social protection program
UCT: unconditional cash transfer
VGD/VGF: vulnerable group development/vulnerable group feeding

## 1 Introduction

Forty percent of households (809 million individuals) in South Asia experience moderate or severe food insecurity, and 30% of children under 5 years old in the region are stunted (FAO, IFAD, UNICEF, WFP, WHO 2023). It is well‐known that nutrition interventions alone will not eradicate poor nutrition outcomes; solutions from multiple sectors that target underlying and immediate determinants of these outcomes are needed (Keats et al. 2021; Bhutta et al. 2013). Cash or in‐kind transfers offered through social protection programs (SPPs), while not necessarily designed with nutrition outcomes in mind, can address underlying determinants missed by nutrition interventions and, in some cases, have been effective at reducing undernutrition among women and children in South Asian contexts (Gentilini 2022). Making SPPs more “nutrition‐sensitive” by adding features that may enhance program impacts on nutrition outcomes has been recommended (Ruel and Alderman 2013; Ruel et al. 2018). For example, a cash transfer may provide relief to a household's budget but without an additional intervention such as information about consuming nutritious foods, the transfer may not lead to impacts on diet and nutrition, as was observed in a trial in Bangladesh (Ahmed et al. 2025). A review of SPPs in South Asia suggests that there is ample scope for making existing programs more nutrition‐sensitive through targeting vulnerable population groups, including nutrition behavior change linking the program to health services, empowering women, providing or ensuring access to foods with a high nutritional value, or including other measures to promote healthy diets (Scott et al. 2023).

While it is important to track the reach of SPPs over time, population‐based multi‐topic household surveys have limited data on exposure to SPPs and their potentially nutrition‐sensitive features (Neupane et al. 2022a). Thus, assessing coverage of nutrition‐sensitive SPPs is currently hindered by poor data availability and measurement challenges. To understand if individuals are reached simultaneously by multiple interventions, estimated indicators of combined coverage or co‐coverage (i.e., the percentage of households receiving more than one intervention of interest) can be generated. Co‐coverage has been used to understand simultaneous coverage of multiple health/nutrition interventions within the same individual/household (Menon et al. 2019; Hong Nguyen et al. 2022; Victora et al. 2005). A non‐representative phone survey in India found low co‐coverage of food and cash transfers with health/nutrition interventions among mothers (Nguyen et al. 2024a); and co‐coverage of SPPs and social and behavior change communication interventions has been shown to be strongly associated with diet quality (Nguyen et al. 2024b). Another study found additive effects of multiple interventions to improve breastfeeding in Vietnamese mothers (Nguyen et al. 2016).

In the current study, using data from representative population‐based surveys that measure household exposure to SPPs, we estimate the co‐coverage of SPPs with health/nutrition interventions during pregnancy and childhood in Bangladesh, India, and Nepal. The co‐coverage estimates provide evidence to identify the gaps and opportunities for improving coverage of interventions for the same target populations delivered by multiple sectors.

## 2 Methods

### 2.1 Data Sources and Classification of SPPs

Secondary data analysis was conducted using multiple publicly available, nationally representative surveys with data on SPPs in three countries: (1) Bangladesh Household Income and Expenditure Survey (HIES) 2016; (2) Bangladesh Multiple Indicator Cluster Survey (BMICS) 2019; (3) India National Family Health Survey (NFHS) 2016; (4) India National Sample Survey (NSS) 2012; (5) Nepal Demographic and Health Survey (NDHS) 2016; (6) Nepal Multiple Indicator Cluster Survey (NMICS) 2019. Different survey data from each country were used to capture different interventions, since a single survey does not capture all interventions. While population‐based surveys in other South Asian countries exist, they were not included in this study due to being older than 15 years, limited accessibility, or not having data on SPPs. The objective was not to present coverage estimates in each country using the most recent representative data, but to explore whether existing data from routine surveys can be used to provide information on co‐coverage of multiple SPP and health/nutrition interventions. The World Bank ASPIRE framework (16) was used to organize the SPPs into five categories involving cash and food transfers—cash for work, unconditional cash transfers (poverty‐targeted cash, allowances, and public charity), conditional cash transfers, food transfers, and school feeding. In these six surveys, data from women with children under 5 years old were used.

### 2.2 Estimating Coverage of SPPs

Coverage for each SPP was defined as the percentage of households with individuals who reported receipt of government cash or food transfers in the past 12 months. For three SPPs targeting pregnant women (*Janani Suraksha Yojana* cash transfer in India, food supplements under India's Integrated Child Development Services (ICDS), and *Aama Karyakram* cash transfer in Nepal), coverage was calculated among women with a pregnancy within the past 5 years. After reporting coverage of each SPP, we summarized the percentage of households covered by any SPP that provided cash and/or food (four aggregate indicators: any cash, any food, any cash or any food, any cash and any food). For example, if there were four SPPs with cash transfers measured in a given survey, the “any cash” coverage indicator was the percentage of households that received transfers from at least one of those SPPs. If there were four cash SPPs and one food SPP measured in a survey, the “any cash and any food” indicator was the percentage of households covered by at least one cash SPP and the food SPP.

### 2.3 Estimating Co‐Coverage of SPPs and Health/Nutrition Interventions

Co‐coverage was defined as receiving both a SPP transfer and key health/nutrition interventions at three different life stages typically noted as being nutritionally vulnerable periods: during the last pregnancy, following delivery, and during early childhood under 5 years of age. The four health/nutrition interventions considered during pregnancy were (1) at least four antenatal care (ANC) visits, (2) any iron and folic acid IFA tablets received, (3) any deworming tablets consumed, and (4) two or more tetanus injections. These same four interventions were used for co‐coverage of interventions with a cash transfer after delivery. For early childhood, the five health/nutrition interventions considered, based on data availability, were: (1) vitamin A supplementation in the past 6 months, (2) deworming tablet received in the past 6 months, (3) iron syrup received in the past 7 days, (4) any growth monitoring received, and (5) any counseling on nutritional status after growth monitoring. For each survey, co‐coverage of the SPP and each health/nutrition intervention was estimated, followed by the target scenario of co‐coverage of the SPP and all health/nutrition interventions combined. For example, the first co‐coverage indicator was the percentage of women with children under 5 years who received any food transfer during pregnancy and at least four ANC visits during the last pregnancy. The second co‐coverage indicator was the percentage of women with children under five who received any food transfer during pregnancy plus any IFA tablets during the last pregnancy, and so forth. Similar co‐coverage indicators were estimated for food plus deworming and food plus tetanus injections. The fifth and final co‐coverage indicator was for an optimal scenario: the percentage of women who received the food transfer plus all four health/nutrition interventions during their pregnancy. The same approach was taken for cash transfers after delivery and food transfers during early childhood. Data were not available for cash transfers during pregnancy, food transfers after delivery, or cash transfers during early childhood in all three countries. Data on health/nutrition interventions were available for India (NFHS 2016) and Nepal (NDHS 2016) but not for Bangladesh surveys. Survey weights were used for all surveys to adjust for differences in the probability of being selected between primary sampling units.

## 3 Results

### 3.1 Description of Survey Data on SPPs and Sample Characteristics

In Bangladesh, coverage data were available for 11 and 6 SPPs, respectively, in HIES 2016 and BMICS 2019 (Table 1). In India, data were available for 2 SPPs in both NSS 2012 and NFHS 2016. In Nepal, data were available only for a maternal conditional cash transfer program in NDHS 2016 and for 5 SPPs in NMICS 2016. No surveys had data on public charity programs. Included programs are listed in Table 1, with additional details for each program provided in Supplementary Table 1. The total number of households included in the surveys ranged from 11,040 in NDHS 2016 to 601,509 in NFHS 2016, and 20%–40% of households had children under 5 years of age (Supplementary Table 2). Supplementary Table 2 also provides additional household characteristics.

**Table 1:** Data availability on social protection programs in population‐based surveys in Bangladesh, India, and Nepal.^a^

Program subcategory	Bangladesh			India			Nepal		
	Data availability		Program name(s)	Data availability		Program name(s)	Data availability		Program name(s)
	HIES2016	BMICS2019		NSS 2012	NFHS2016		NDHS 2016	NMICS2019	
Cash for work	**P**	**P**	Work for money; test relief; employment generation program for the poorest	**O**	**O**	The Mahatma Gandhi National Rural Employment Guarantee	**O**	**O**	Prime Minister Employment program
UCT									
Poverty‐targeted cash	**P**	**O**	Targeted ultra‐poor program	NA	NA	—	**O**	**P**	Endangered Indigenous Peoples Allowance or Endangered Ethnicity Grant
Allowances									
Old age	**P**	**P**	Old‐age allowance program	**O**	**O**	Indira Gandhi National Old Age Pension Scheme	**O**	**P**	Old Age Allowance or Senior Citizen's Allowance
Widow/single women	**P**	**P**	Husband‐deserted; Widowed and Destitute Women Allowance	**O**	**O**	Indira Gandhi National Widow Pension Scheme	**O**	**P**	Single Women's Allowance
Disabled	**P**	**O**	Allowance for Financially Insolvent Persons with Disabilities	**O**	**O**	Indira Gandhi National Disability Pension Scheme	**O**	**P**	Disability Grant
Martyr's^b^	**P**	**P**	Allowance for freedom fighters/Shaheed family	NA	NA	—	—	—	—
Public charity	NA	NA	—	NA	NA	—	NA	NA	—
CCT									
Women/pregnant women	**P**	**P**	Maternity allowance program for the poor lactating mothers	**O**	**P**	Janani Suraksha Yojana	**P**	**O**	Aama program
Students	**P**	**O**	Primary Education Stipend program; Secondary Education Sector Investment program; Anand School program	NA	NA	—	**O**	**O**	Scholarships program
Food transfer	**P**	**P**	Vulnerable group development/vulnerable group feeding	**P**	**P**	Food supplements to pregnant and lactating women and children under integrated Child Development services program	**O**	**O**	Supplementary food for lactating women and children in food insecure areas
Nutrition program^c^	**P**	**O**	Improving Maternal and Child Nutrition; Community Nutrition program	NA	NA	—	**O**	**P**	Child (0‐5 years) grant
School feeding	**P**	**O**	School feeding program in the poverty‐prone areas	**P**	**O**	Mid‐day meal	**O**	**O**	National School Meals program; Food for Education

Abbreviations: CCT, conditional cash transfer; HIES, Household Income and Expenditure Survey; MICS, Multiple Indicator Cluster Survey; NDHS, Nepal Demographic and Health Survey; NFHS, National Family Health Survey; NSS, National Sample Survey; UCT, unconditional cash transfer.

^a^ For a more detailed description of each program, refer to Supplementary Table 1.

^b^ Family of a person who died in a war.

^c^ Any transfer (cash or food) that may be linked to the fulfillment of nutrition practices, such as completing growth monitoring.

**P** = Program and data available; **O** = Program available but data not available; NA = Program does not exist, thus data availability assessment was not applicable.

### 3.2 Coverage of SPPs and Health/Nutrition Interventions

Coverage of SPPs in Bangladesh and Nepal was less than 20% in nearly all SPPs and less than 5% in many SPPs, and about 50% for SPPs in India (Table 2). Between 12% (Bangladesh) and 41% (India) of households received at least one cash transfer program. Coverage of at least one food transfer program was highest in India, where 60% of households received take‐home rations through the ICDS program. In Bangladesh, only 3% of households received a food transfer through the Vulnerable Group Development program or a school feeding program per HIES 2016 data. In Bangladesh, 18% of households received either cash or food but less than 2% received both cash and food. In India, due to moderate coverage of Janani Suraksha Yojana and of take‐home rations through ICDS, 16% of households received both cash and food.

**Table 2:** Coverage of individual social protection programs in the past 12 months in Bangladesh, India, and Nepal.^a^

	Bangladesh		India		Nepal	
	HIES 2016	BMICS 2019	NSS 2012^d^	NFHS 2016	NDHS 2016	NMICS 2019
	*%*	*%*	*%*	*%*	*%*	*%*
Social protection programs						
Cash for work						
Employment generation	—	1.2	—	—	—	—
Gratuitous relief	1.4	—	—	—	—	—
Test relief	0.6					
Unconditional cash transfer						
Old age allowance	4.1	—	—	—	—	12.9
Widow allowance	1.3	—	—	—	—	6.8
Disabled allowance	0.4	—	—	—	—	1.3
Any unconditional allowances^b^	—	10.2	—	—	—	—
Conditional cash transfer						
Anand school program	0.4					
Stipend for primary school students	6.9	—	—	—	—	—
Stipend for secondary school students	2.7	—	—	—	—	—
Maternity allowance	—	0.8				
Janani Suraksha Yojana^c^	—	—	—	41.1	—	—
Aama Karyakram^c^	—	—	—	—	86.4	—
Nutrition program						
Child support grant	—	—	—	—	—	2.9
Food support						
VGD/VGF	2.5	7.4	—	—	—	—
Public distribution system	—	—	44.1	—	—	—
Food supplement for pregnant women under ICDS^c^	—	—	—	51.9	—	—
Food supplement for children under ICDS^c^				51.3		
School feeding	0.9	—	37.9	—	—	—
Transfer type received (aggregate indicators)						
Any cash	15.9	11.6	—	41.1	86.4	22.1
Any food	3.3	7.4	51.8	59.8	—	—
Any cash or any food	18.3	17.7	—	65.9	—	—
Any cash and any food	1.0	1.6	—	15.7	—	—
Nutrition/health interventions						
Pregnancy						
≥ 4 ANC visits, %	—	—	—	49.3	69.4	—
Any IFA, %	—	—	—	77.7	90.9	—
Deworming, %	—	—	—	18.0	70.2	—
Two tetanus injections, %	—	—	—	83.5	66.1	—
Early childhood						
Vitamin A in past 6 m (6–59 m), %	—	—	—	57.8	86.0	—
Deworming in past 6 m (12–59 m), %	—	—	—	33.0	75.8	—
Iron syrup in past 7 days (6–59 m), %	—	—	—	26.0	7.7	—
Growth monitored (0–59 m), %	—	—	—	43.3	25.3	—
Counseling after growth monitoring (0‐59 m), %	—	—	—	27.8	12.2	—

Abbreviations: BMICS, Bangladesh Multiple Indicator Cluster Survey; HIES, Household Income and Expenditure Survey; ICDS, Integrated Child Development Service; NDHS, Nepal Demographic and Health Survey; NFHS, National Family Health Survey; NMICS, Nepal Multiple Indicator Cluster Survey; NSS, National Sample Survey; VGD, Vulnerable Group Development; VGF, Vulnerable Group Feeding.

^a^ For a more detailed description of each program, refer to Supplementary Table 1.

^b^ BMICS 2019 asked about all allowances combined (old age/disabled/widow/freedom fighters/shared families, etc.).

^c^ The recall period for these programs was the previous pregnancy (not necessarily the last 12 months).

^d^ In India, NSS data is shown for the percentage of households that received rice or wheat in the past 30 days or with children 6–14 years old who received a free school meal in the past 30 days.

Coverage of health and nutrition interventions during pregnancy was lower in India compared to Nepal, except for tetanus injections. There was a large variation in coverage of interventions during early childhood, ranging from 26% (IFA syrup) to 58% (vitamin A supplementation) in India and from 8% (IFA syrup) to 86% (vitamin A supplementation) in Nepal.

### 3.3 Co‐Coverage of SPPs and Health/Nutrition Interventions

**Figure 1:**
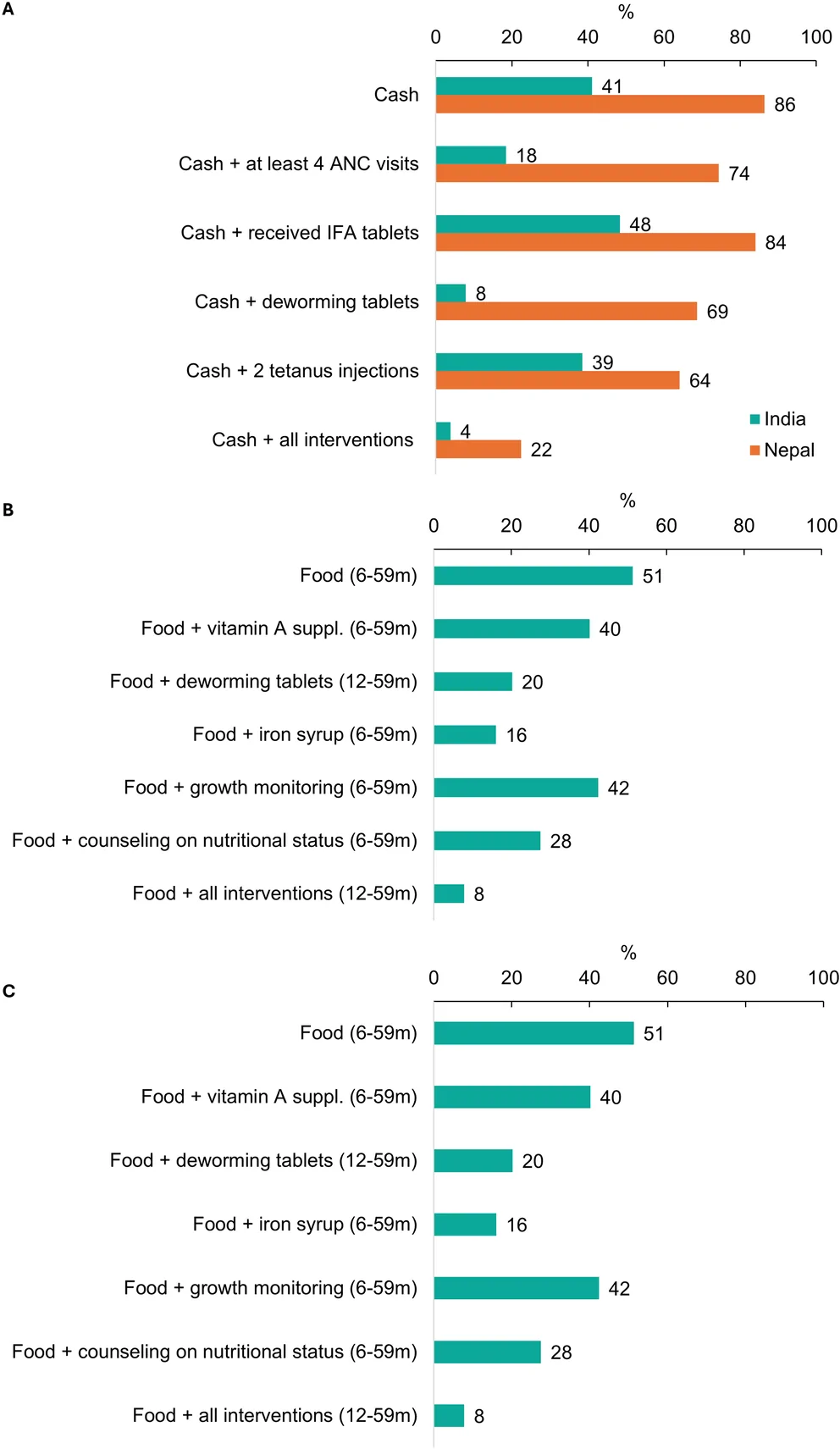
Co‐coverage of transfers with other health/nutrition interventions during pregnancy (A), after delivery (B) or in early childhood (C) in India and Nepal. Food in India's NFHS 2016 refers to food supplementation during pregnancy and during early childhood under the Integrated Child Development Services program; the denominator for this analysis was women who gave birth in the past 5 years for women and denominators varied by interventions for children according to age group. Cash in India's NFHS 2016 refers to cash received through Janani Suraksha Yojana. Cash in Nepal's NDHS 2016 refers to cash received through Aama Karyakram. ANC = antenatal care, IFA = iron folic acid, NDHS = Nepal Demographic and Health Survey, NFHS = National Family Health Survey.

Among the six surveys, only NFHS 2016 and NDHS 2016 had data on both SPPs and health/nutrition interventions. During their last pregnancy, half (52%) of women in India with a birth in the past 5 years received a food transfer, a take home ration through the ICDS scheme, and only 3% received a food transfer plus four health/nutrition interventions: having at least four ANC visits, receiving any IFA tablets, receiving deworming tablets, and receiving two tetanus toxoid injections (Figure 1A). Similarly, in India and Nepal, respectively, while 41% and 86% of women received cash through perinatal cash transfer programs, only 4% and 22% of women received cash plus all four health/nutrition interventions (Figure 1B). Co‐coverage of India's ICDS food transfer and health/nutrition interventions during early childhood followed the same pattern, with 51% of children getting the transfer but only 8% of children getting the transfer plus all five health/nutrition interventions (Figure 1C).

## 4 Discussion

While many SPPs and health/nutrition data systems exist in South Asia (Neupane et al. 2024, 2022b), measurements of whether households and/or target populations receive benefits and related program features in population‐based surveys are often lacking or capture partial information (Gillespie et al. 2019). Using readily available data from three South Asian countries, we observed that few surveys measured coverage of both SPPs and health/nutrition interventions simultaneously. Despite the existence of SPPs in these countries, routine multitopic household surveys often do not collect information on receipt of benefits from their programs. Surveys from Bangladesh had the most data on SPPs, but nutrition/health interventions were not measured. Surveys that did measure coverage of nutrition/health interventions (e.g. NFHS in India and NDHS in Nepal) had little data on coverage of SPPs. Where data on both SPPs and health/nutrition interventions were available, we found poor overlap of multiple interventions in the same households. These findings point to a missed opportunity to reach households with both SPP and health/nutrition interventions.

Many examples of large‐scale SPPs exist in South Asia, but they are infrequently and inadequately measured. Although social protection systems and, thus, SPPs differ across countries, certain SPPs that address underlying determinants of malnutrition, such as school feeding programs or perinatal cash transfers, exist across contexts in South Asia. Therefore, measuring both common SPP elements and generic features proven to improve nutrition outcomes through a standard set of questions in household surveys would enable cross‐country comparisons of SPP coverage. Questions that ask about additional details on the transfers (e.g. quantity of cash received or types of food received as part of school meals), would allow for further program refinement and exploration of how program quality relates to measured nutrition outcomes. In the absence of such data on the potential health and nutrition benefit features of SPPs that are designed to be nutrition‐sensitive, co‐coverage is a useful approach to examine if households are receiving multiple interventions, but the feasibility of co‐coverage estimation remains limited due to the few surveys measuring receipt of both SPPs and health/nutrition interventions.

Our co‐coverage estimates can be compared to those from a phone survey in India conducted during the COVID‐19 pandemic that included detailed questions on receipt of SPPs and health/nutrition interventions by women (Nguyen et al. 2024a). Co‐coverage estimates in the phone survey were higher than what we found in the current study, possibly because the phone survey was more recent (2022 compared to 2016 for NFHS), thus existing programs had wider reach, and there were new programs; the phone survey also included a broader set of SPPs and health/nutrition interventions compared to NFHS. The current work was an illustrative analysis, and we acknowledge that replicating the analysis with other or more recent datasets may lead to alternative co‐coverage estimates. There are modifications between each round of DHS, for example, and additional SPPs or nutrition interventions could be measured. When modifying surveys, close attention should be paid to recall periods to allow for comparability across surveys, programs, and countries as much as possible.

Many non‐governmental organizations provide social protection services in focused geographies, which are often not tracked in population‐based surveys. In any given survey, there is a limitation to the amount of information that can be collected; thus multiple data sources should be utilized when possible. SPP coverage estimates from nationally representative surveys such as DHS and MICS may not account for all existing SPPs within a country. Nonetheless, our findings reveal gaps and opportunities across life stages for tracking interventions. For example, in Bangladesh, MICS surveys which already capture receipt of cash/food transfers could integrate questions on key nutrition/health interventions, including nutrition education and habits. Some countries have administrative data systems that track social assistance disbursed to beneficiaries as well as services across sectors. Nepal's Multi‐Sector Nutrition Plan‐III (2023–2030) promotes an integrated nutrition information management system to track progress across health, education, agriculture, water and sanitation, and social protection sectors (Government of Nepal NPC 2024). A challenge of many administrative data systems, however, is that only those who access the service are recorded in the system, thus coverage estimates may be underrepresented, especially for vulnerable groups, unless specific outreach and inclusion mechanisms are in place. Thus, household surveys remain critical for assessing equitable co‐coverage, i.e. which types of individuals or households are missed by programs, which was not a focus of the current analysis. It is also worth noting that overlap of programs indicated by high co‐coverage does not necessarily reflect strong coordination or integration across sectors; household may receive services independently rather than through deliberate systems convergence. Future research should triangulate information from multiple data sources to more closely examine SPP coverage and co‐coverage of multisectoral interventions.

SPPs or health/nutrition interventions in isolation have a limited scope to respond to the broad range of shocks and challenges that households face during critical periods of growth and development. Ensuring that vulnerable households are reached by complementary interventions that provide both social protection transfers and health/nutrition interventions to address underlying and intermediate needs involves multiple challenges (Avula et al. 2015) but is critical for optimal maternal, infant and young child wellbeing. Opportunities to strengthen co‐coverage measurement include integration of standardized SPP and health/nutrition indicators into multitopic surveys, improving interoperability between administrative and survey datasets, routine tracking of convergence indicators across sectors, and incorporation of nutrition‐sensitive dimensions within SPP monitoring systems. Measurement of SPPs, their nutrition‐sensitive features, and health/nutrition interventions in population‐based surveys requires further attention and investment.

## Author Contributions

S.S., S.N., and S.S.K. designed the study, conducted the analysis, and drafted the initial manuscript. H.T., N.A., H.A., and R.H. provided substantial contributions to the interpretation of data and reviewed and provided comments on the drafts. B.C. conducted a literature review and contributed to drafting the manuscript. All authors reviewed and approved the final manuscript.

## Conflicts of Interest

The authors declare no conflicts of interest.

## Supporting information


Supporting File

## Data Availability

The data that support the findings of this study are available in online repositories at https://microdata.worldbank.org/. These data were derived from the following resources available in the public domain: ‐ International Household Survey Network, https://catalog.ihsn.org/catalog/7399/study-description ‐ World Bank, https://microdata.worldbank.org/index.php/catalog/4147 ‐ Government of India Ministry of Statistics, https://microdata.gov.in/NADA/index.php/catalog/126 ‐ World Bank, https://microdata.worldbank.org/index.php/catalog/2949 ‐ World Bank, https://microdata.worldbank.org/index.php/catalog/2929 ‐ World Bank, https://microdata.worldbank.org/index.php/catalog/4167.
